# Evaluation of an evidence-based medicine educational intervention in a regional medical campus

**Published:** 2018-03-27

**Authors:** Mylene Lévesque, Janie Gauthier-Boudreau, Paul Gagnon, Bastian Bertulies-Esposito, Sharon Hatcher, Louis Gagnon

**Affiliations:** 1 Centre intégré universitaire de santé et de services sociaux du Saguenay-Lac-Saint-Jean, Québec, Canada; 2 Université du Québec à Chicoutimi, Québec, Canada; 3 Univerisité de Montréal, Québec, Canada; 4 Univerisité de Sherbrooke, Québec, Canada

## Abstract

**Background:**

Enhanced educational activities were developed by a regional medical campus (RMC) in order to incorporate evidence-based medicine (EBM) practice in the learning process of medical students. This study aimed to measure the effectiveness of these activities.

**Methods:**

The experimental group was made up of third-year students from the RMC. The comparison group included students from the main campus of the medical school and another of its RMCs. The experimental group received additional training on EBM: one additional hour in class, plus skills development exercises throughout the semester. During the regular academic sessions, clinical questions requiring EBM literature searching skills were incorporated in the curriculum. Tests on knowledge and self-assessment of competencies were administered to all participants at the beginning and at the end of the semester. Data were analyzed using repeated measures analysis of variance and post hoc tests for within and between groups comparison.

**Results:**

The Friedman test demonstrated a statistically significant effect of the intervention on knowledge (p <0.0001). The score of the knowledge test was significantly higher for the experimental group, when compared with baseline testing and with the comparison group (p <0.0001). Repeated measures analysis of variance demonstrated a statistically significant effect of the intervention on the score of the self-assessment of competencies (p=0.032). The score for the self-assessment of competencies was significantly higher for the experimental group when compared to baseline score (p <0.0001), but not with respect to the comparison group.

**Conclusion:**

Our study demonstrated the effectiveness of additional training and longitudinal integrated skills development leading to an increase in medical student knowledge and self-perception of competencies in EBM practice.

## Introduction

The establishment of a regional medical campus (RMC) provides the opportunity to define its own mission relevant to the needs of the population it serves. It also allows flexibility to develop specific aspects of the curriculum targeted to the needs of its future graduates in order to practice in sites distant from tertiary care medical centers.^1^,^2^ Evidence-based medicine (EBM) is one of these essential elements of practice becoming part of medical curricula around the world. North American and Canadian stakeholders in medical education have made EBM practice a core competency to develop.^3^,^4^ Thus, the Saguenay Medical Education Program (based in an RMC of the Université de Sherbrooke, Quebec, Canada) decided to include the development of EBM competencies in its mission statement, and to conceive a pilot project with enhanced EBM learning activities in the pre-clerkship medical curriculum, in order to better prepare its students for the needs of regional medical practice.

EBM is defined as: “The conscientious, explicit, and judicious use of current best evidence in making decisions about the care of individual patients.”^5^ There are five critical steps one needs to follow in order to practice EBM: 1) construct a question from the clinical environment; 2) search the medical literature to identify the best available evidence; 3) appraise the evidence; 4) integrate the evidence in clinical practice; and 5) evaluate its application.^6^

Many studies have evaluated the integration of EBM in medical curricula.^7-10^ Integration of EBM benefits medical students with regard to their attitudes and skills practicing EBM.^7-9^,^11^ Different strategies have been used to integrate the practice of EBM in medical curricula, but the exact amount of sessions and content vary widely across studies.^12-19^ One study demonstrated that a single session to practice EBM is not sufficient to acquire real competencies.^20^ Training in EBM practice has resulted in improvement of student literature searching skills in all cases,^21-24^ but educational activities more integrated in the curriculum showed more effectiveness in student acquisition and use of those skills.^25^,^26^ It is worth noting that few studies employed a comparison group.^21^

Many obstacles remain for the integration of EBM practice in medical education and clinical practice. Medical students do not necessarily see EBM practice as being relevant to their clinical work.^11^ Another challenge in the teaching of EBM is enabling students to correctly and efficiently search and retrieve information.^27-30^ The PICO method, which stands for: P (Populations/People/Patient/Problem); I (Intervention); C (Comparison); O (Outcome), has been proven to be efficient and easy to use, as it allows students to correctly transfer a clinical situation into an answerable question that fits literature search engines, leading to more efficient searches.^31-34^ The PICO method helps identification and organization of key concepts related to a given clinical problem. Its use was incorporated longitudinally in the Saguenay pilot project aimed at enhancing EBM training in a problem-based learning (PBL) curriculum. This study aimed to evaluate the impact of the integration of skills development activities using a PICO approach on student knowledge and perception of competencies related to EBM practice.

## Methods

### Participants

Participants were third-year medical students of two successive cohorts, recruited at the beginning of their last semester of preclinical training in the Université de Sherbrooke MD Program. The University has three academic sites, one main campus in Sherbrooke, and two RMC sites, one in Saguenay and one in Moncton, New Brunswick. The total student population is on average 200, and is unequally divided among the three campuses (see Table 1). As admission criteria and the curriculum are the same for the three campuses, the characteristics of the students with respect to variables such as age, gender, and previous schooling were known to be similar between all sites.

**Table 1:** Population and sampling of students (number of students)

Group		Comparison		Experimental

Campus		Moncton	Sherbrooke	Saguenay

**Total population of students**	**Cohort 1**	23	148	33
	**Cohort 2**	25	141	31

	**Total**	337		64

**Students invited to participate**	**Cohort 1**	23	50	33
	**Cohort 2**	25	50	31

	**Total**	108		64

**Participating students**	**Cohort 1**	10	29	31
	**Cohort 2**	25	17	30

	**total**	81		61

### Instruments

Two instruments were used to evaluate students. Knowledge was evaluated using a French adaptation of the Fresno questionnaire.^9^ This questionnaire, developed by the University of California in San Francisco, and possesses good psychometric properties.^10^ It is designed to evaluate the level of knowledge in EBM literature searching skills. The translated and adapted version used in the study was not formally validated for feasibility reasons, but was based on a valid and reliable questionnaire.^7^ The translation was made by team members and each question was discussed. It had 11 questions with multiple choice or short answer responses totaling 70 points. Questions had to be answered in 30 minutes or less.

Students reported their perception of their competencies using a self-assessment questionnaire developed by the study team. The questionnaire was based on the competent, information literate student from the standards of the American Association of College Research Libraries:^3^ 1) Determines the nature and extent of the information needed; 2) Accesses needed information effectively and efficiently; 3) Evaluates information and its sources critically and incorporates selected information into his or her knowledge base and value system; 4) Uses information effectively, individually or as a member of a group, to accomplish a specific purpose; 5) Understands many of the economic, legal, and social issues surrounding the use of information and accesses and uses information ethically and legally. Some recent studies suggest that group self-assessment is an acceptable measure for the evaluation for educational programs.^35,36^ Moreover, the research team wanted to evaluate the confidence of students in applying their knowledge of EBM practice. The questionnaire contained 24 questions answered by a Likert-type scale ranging from 0 to 10 (0=not competent, 10=very competent).

### Intervention

The study took place between September 2013 and December 2014, using two cohorts of third-year students. In September 2013 and 2014, students in each of the three academic sites were solicited to participate in the study. To have an equivalent number of students participating in the study from each campus, all the third-year students in both RMCs were contacted, whereas only a sample of the student population in the main campus (Sherbrooke) was selected randomly for the study. Table 1 presents the different student samples in each campus and for each cohort that were invited to participate in the study. The students from the Saguenay RMC formed the experimental group whereas the comparison group was composed of the students from the main campus plus the students from the Moncton RMC. The study was presented to the students during a class, before the EBM course in each campus. Students who agreed to participate were invited to complete as a baseline the Fresno knowledge test and the self-assessment of competencies prior to attending a two-hour lecture on literature searching skills.

The experimental group received an additional hour of training on the PICO method^37^ and additional information on scientific literature searches such as a demonstration of how to use some databases that were not included in the comparison group lecture. Thereafter, students in the experimental group were asked to answer predetermined clinical questions requiring an EBM approach that were integrated in the small group clinical reasoning sessions employed in the curriculum through-out the semester. There were ten clinical questions to answer for the first cohort of students, and seven questions for the second cohort. At the end of the first year, the research team met with students to receive their feedback on the pilot project. Students found that there were too many questions as they had experienced some redundancy in the use of databases and the type of search they had to do. The learning activities were thus adjusted by reducing the number of questions. As the small learning groups met to discuss the subject covered in the clinical problem of the week, teachers were asked to select a student who would explain the techniques he or she used to search the literature in order to answer the predetermined clinical question. The following is one of the clinical questions: “Does a multidisciplinary team intervention have an impact on patient readmission and mortality for patients with heart failure?” Using the PICO method, students had to find key words and create a search strategy to use in a medical literature database. With the results from their searches, they had to read abstracts and try to find an answer to the clinical question in scientific articles. At the end of the semester, the same tests on knowledge and self-assessment of competencies were administered to all participants.

Ethics approval was obtained from the Comité d’éthique de la recherche – éducation et sciences sociales de l’Université de Sherbrooke.

### Statistical analysis

Results are presented as the mean ± standard deviation. Normality was assessed using the Shapiro-Wilk test on the standardized residuals. The repeated measures analysis of variance (RM-ANOVA) or the Friedman tests were conducted to test the effect of the intervention on test scores, both for scores of the knowledge test and the self-assessment of competencies. After these analyses, the results of the different tests were compared between the experimental and comparison groups, using the *t*-test or the Mann-Whitney test when appropriate. Within group analyses were also performed using the paired *t*-test or the Wilcoxon test when appropriate. Data were analyzed using SPSS 20.

## Results

### Participants

Table 2 presents the number of participants per group who completed pre and post evaluations for each questionnaire, and whose test scores served as the basis for statistical analysis. Fifty-nine participants in the experimental group and 65 participants in the comparison group completed the knowledge test. The self-assessment of competencies was answered by 27 participants in the experimental group and 55 participants in the comparison group.

**Table 2:** Population and sampling of students (number of students)

Group		Comparison		Experimental
Campus		Moncton	Sherbrooke	Saguenay
**Knowledge test**	**Participants per site**	20	35	59
	**Total**	65		59
**Self-assessment on perception of competencies**	**Participants per site**	22	33	27
	**Total**	55		27

### Knowledge test

The Friedman test demonstrated a statistically significant effect of the intervention on knowledge (χ2= 47.15; p<0.0001). Comparisons were made between initial and final tests for each group and between initial and final tests between groups (Table 3). There was a significant difference between the initial and final testing for the experimental group, and between the experimental and comparison groups for final testing (p<0.0001). However, there was no difference in the comparison group between the initial and final test.

**Table 3:** Mean ± standard deviation, intra-group and inter-group comparison for the knowledge test (maximum score=70)

Group	Participants	Initial test	Final test	Statistics and p
**Experimental**	59	37.10±7.74	47.53±9.36	W = 1603 p<0.0001
**Comparison**	65	38.38±7.82	40.06±10.09	W = 1231.5 p = 0.074
**Statistics and p**		t = -0.92 p = 0.361	U = 1040.5 p<0.0001	

### Self-assessment of competencies test

Levene’s test was used to assess the homogeneity of variance as it is required for RM-ANOVA. The equality of variance could not be rejected for the pre-test and the post-test (F=0.072, p=0.789; F=1.471, p=0.229). RM-ANOVA demonstrated a statistically significant effect of the intervention on the score of the self-assessment of competencies test (F= 5.160; p=0.032). Comparisons were made between initial and final tests for each group and between initial and final tests between groups (Table 4). There was a significant difference between the initial and final self-assessments for the experimental group (p<0.0001). There was no significant difference in the final self-assessments between the experimental group and the comparison group (p>0.05).

**Table 4:** Mean ± standard deviation, intra-group and inter-group comparison for the self-assessment of competencies test (maximum score=240)

Group	Participants	Initial test	Final test	Statistics and p
**Experimental**	27	135.04±32.74	168.68±26.20	t = -5.55 p < 0.0001
**Comparison**	55	162.72±29.80	166.30±31.06	t = -0.68 p = 0.501
**Statistics and p**		t = -3.83 p < 0.0001	t = 0.34 p = 0.733	

## Discussion

The aim of this study was to evaluate the impact of an integrated EBM skills development project in the third year of a medical curriculum. The effect of the intervention was quantified using two instruments and compared between two groups. Overall, the intervention had a positive effect on knowledge and self-perception of competencies for students in the experimental group.

The score of the knowledge test was significantly higher at final testing for the experimental group when compared to the comparison group, and when compared to itself at baseline testing. Thereby, the students in the experimental group increased their knowledge of EBM with the training sessions, and also gained more knowledge than students who did not receive the same training. The students in the comparison group did not significantly increase their knowledge when compared to themselves at baseline testing, confirming the positive effect of the enhanced longitudinal training on knowledge of EBM. Other studies have demonstrated that longitudinal integration of EBM competencies in medical curricula produce a notable change in Fresno test scores.^9^,^11^ West et al. obtained an increase of 26% in Fresno test scores after integration of EBM training, and Aronoff et al. obtained a 9% increase.^8,38^ The results of this study showed a 15% increase in the knowledge test. The differences among studies could be explained by methodological variance. In the West et al. study, EBM practice was integrated in the second year of the curriculum, whereas Aronoff’s study integrated EBM only in the third year, through an online course.

Results from the self-assessments were consistent with results from the knowledge test, suggesting that students from the experimental group gained more knowledge, but also felt more comfortable and competent to use their newly acquired skills. Results demonstrated that students in the experimental group increased their perception of their capacity when compared to themselves at baseline, but not when compared to the comparison group. With regard to the difference between scores at the beginning of the study, where the comparison group scored higher than the experimental group at baseline, it is plausible that the implementation of the project itself influenced student self-assessment. The students in the experimental group were more aware of their lack of knowledge in EBM practice than those of the two other sites forming the comparison group, possibly inducing a bias in their self-assessment.^39^ Also, the presence and the support of the research team in the Saguenay site could have benefited the experimental group. The same support was available in the other campuses, but students could have been less sensitized to librarian services. Results were still consistent with the literature. Studies have shown that students who followed a longitudinal curriculum of EBM practice reported having better attitudes and confidence in their searching skills of evidence-based data.^7^,^40^

Our study has some limitations. The two tools that were used to evaluate students have not been formally validated. It was not possible for the research team to find French validated questionnaires or to perform this validation given that time and resources were limited. Nevertheless, questionnaires were developed based on scientific and validated materials.^3^,^10^ For the knowledge test, the team adapted and translated into French the Fresno questionnaire which is valid and reliable39 in English.^7^ The translation was made by team members and each question was discussed. The self-assessment of competencies test was based on the Information literacy competency standards for higher education, which has been developed by the American Association of College and Research Libraries.^3^ Another limit could be the use of the pretest as opposed to the use of a then test. According to the literature, self-assessment is a more reliable program evaluation tool when the then and post tests are done separately but both after the intervention. In our study, we separated the self-assessments, but conducting the then test as a pretest can cause a response shift bias.^39^,^41^

Another limitation of this study was the loss to follow-up. Some issues arose during the two years of the study that diminished our final sample size. The knowledge questionnaire was administered in class as it had to be completed in under 30 minutes. For the first cohort, we had to exclude the whole group from the Moncton campus. This is because students were given the test to answer at home, so the equivalent time constraint was not applied as it was for the Saguenay and Sherbrooke groups. This situation was corrected for the second cohort. The pre-test on self-assessment of competencies was sent as an online survey, but students were asked to complete it in class. The post-test was sent online after students had started their clerkship rotations and were no longer in their respective campuses. They were more difficult to reach as they were spread out in various locations. Many email reminders were sent, but the response rate was lower than for the knowledge test. As it was a paired analysis, participants who did not complete both tests were excluded from the analysis. We could not measure the possible difference in characteristics between participants who responded and those who did not. However, we can hypothesize that participants who were better or more motivated tended to respond more than others. However, this bias can be true for all three sites and, therefore, cannot explain the differences found in the study.

### Conclusion

This study highlighted the capacity of RMCs to develop and implement small-scale innovative educational projects and to evaluate their effectiveness. The study demonstrated that the educational intervention used to integrate EBM learning in a third-year medical curriculum at an RMC was effective as students gained knowledge about this topic and were more confident to use their EBM skills. It also demonstrated that longitudinal integrated learning activities were more effective in acquiring knowledge and mobilizing student EBM competencies. Hopefully, this study will contribute to ensuring that future physicians be better equipped for EBM practice at whatever site they are trained.

## References

[1] Hatala R, Keitz S, Wilson M, Guyatt G Beyond journal clubs. Moving toward an integrated evidence-based medicine curriculum. J Gen Intern Med. 2006;21:538-41. 16704406 10.1111/j.1525-1497.2006.00445.x PMC1484798

[2] Ferwana M, Alwan I, Moamary M, Magzoub M, Tamim H Integration of evidence based medicine into the clinical years of a medical curriculum. Journal of Family and Community Medicine. 2012;19(2):136-40. 22870419 10.4103/2230-8229.98307 PMC3410178

[3] Association of college and research libraries Information literacy competency standards for higher education. In. Standards, performance indicators, and outcomes. Chicago, Ill: Association of College and Research Libraries; 2000.

[4] Royal College of Physicians and Surgeons of Canada. CanMEDS Framework. 2011 [Internet]. Available at: http://www.royalcollege.ca/portal/page/portal/rc/canmeds/framework#scholar . [Accessed February 26, 2015].

[5] Sackett DL, Rosenberg WM, Gray JA, Haynes RB, Richardson WS Evidence based medicine: what it is and what it isn't. BMJ. 1996;312(7023):71-2. 8555924 10.1136/bmj.312.7023.71 PMC2349778

[6] Ilic D, Tepper K, Misso M Teaching evidence-based medicine literature searching skills to medical students during the clinical years: a randomized controlled trial. Journal Of The Medical Library Association. 2012;100(3):190-6. 22879808 10.3163/1536-5050.100.3.009 PMC3411272

[7] Liabsuetrakul T, Suntharasaj T, Tangtrakulwanich B, Uakritdathikarn T, Pornsawat P Longitudinal analysis of integrating evidence-based medicine into a medical student curriculum. Fam Med. 2009;41(8):585-8. 19724944

[8] West CP, Jaeger TM, McDonald FS Extended evaluation of a longitudinal medical school evidence-based medicine curriculum. J Gen Intern Med. 2011;26(6):611-5. 21286836 10.1007/s11606-011-1642-8 PMC3101983

[9] Barghouti FF, Yassein NA, Jaber RM, et al Short course in evidence-based medicine improves knowledge and skills of undergraduate medical students: a before-and-after study. Teach Learn Med. 2013;25(3):191-4. 23848323 10.1080/10401334.2013.797348

[10] Ramos KD, Schafer S, Tracz SM Validation of the Fresno test of competence in evidence based medicine. BMJ. 2003;326(7384):319-21. 12574047 10.1136/bmj.326.7384.319 PMC143529

[11] Lai N Teaching evidence-based medicine: a clinician’s perspective. Malaysian Family Physician. 2013;8(2):7. 25606275 PMC4170478

[12] Srinivasan M, Weiner M, Breitfeld PP, Brahmi F, Dickerson KL, Weiner G Early introduction of an evidence-based medicine course to preclinical medical students. Journal of general internal medicine. 2002;17(1):58. 11903776 10.1046/j.1525-1497.2002.10121.x PMC1494995

[13] Wanvarie S, Sathapatayavongs B, Sirinavin S, Ingsathit A, Ungkanont A, Sirinan C Evidence-based medicine in clinical curriculum. Ann Acad Med Singapore. 2006;35(9):615-8. 17051277

[14] Okoromah CA, Adenuga AO, Lesi FE Evidence-based medicine curriculum: impact on medical students. Med Educ. 2006;40(5):465-6. 16635133 10.1111/j.1365-2929.2006.02454.x

[15] Weberschock TB, Ginn TC, Reinhold J, et al Change in knowledge and skills of Year 3 undergraduates in evidence-based medicine seminars. Med Educ. 2005;39(7):665-71. 15960786 10.1111/j.1365-2929.2005.02191.x

[16] Dorsch JL, Aiyer MK, Meyer LE Impact of an evidence-based medicine curriculum on medical students' attitudes and skills. J Med Libr Assoc. 2004;92(4):397-406. 15494754 PMC521510

[17] Holloway R, Nesbit K, Bordley D, Noyes K Teaching and evaluating first and second year medical students' practice of evidence-based medicine. Med Educ. 2004;38(8):868-78. 15271048 10.1111/j.1365-2929.2004.01817.x

[18] Shaneyfelt T, Baum KD, Bell D, et al Instruments for evaluating education in evidence-based practice: A systematic review. JAMA. 2006;296(9):1116-27. 16954491 10.1001/jama.296.9.1116

[19] Johnston JM, Leung GM, Fielding R, Tin KYK, Ho Lm The development and validation of a knowledge, attitude and behaviour questionnaire to assess undergraduate evidence-based practice teaching and learning. Medical Education. 2003;37(11):992-1000. 14629412 10.1046/j.1365-2923.2003.01678.x

[20] MacEachern M, Townsend W, Young K, Rana G Librarian Integration in a Four-Year Medical School Curriculum: A Timeline. Medical Reference Services Quarterly. 2012;31(1):105-14. 22289100 10.1080/02763869.2012.641856

[21] Ghali WA, Saitz R, Eskew AH, Gupta M, Quan H, Hershman WY Successful teaching in evidence-based medicine. Med Educ. 2000;34(1):18-22. 10607274 10.1046/j.1365-2923.2000.00402.x

[22] Gruppen LD, Rana GK, Arndt TS A Controlled Comparison Study of the Efficacy of Training Medical Students in Evidence-Based Medicine Literature Searching Skills. Academic Medicine. 2005;80(10):940-4. 16186614 10.1097/00001888-200510000-00014

[23] Vogel EW, Block KR, Wallingford KT Finding the evidence: teaching medical residents to search MEDLINE. J Med Libr Assoc. 2002;90(3):327-30. 12113519 PMC116407

[24] Rosenberg WM, Deeks J, Lusher A, Snowball R, Dooley G, Sackett D Improving searching skills and evidence retrieval. J R Coll Physicians Lond. 1998;32(6):557-63. 9881313 PMC9662986

[25] Del Mar C, Glasziou P, Mayer D Teaching evidence based medicine. In: BMJ. Vol 329 England 2004:989-90. 15514319 10.1136/bmj.329.7473.989 PMC524536

[26] Coomarasamy A, Khan KS What is the evidence that postgraduate teaching in evidence based medicine changes anything? A systematic review. BMJ. 2004;329(7473):1017. 15514348 10.1136/bmj.329.7473.1017 PMC524555

[27] Green ML, Ruff TR Why Do Residents Fail to Answer Their Clinical Questions? A Qualitative Study of Barriers to Practicing Evidence-Based Medicine. Academic Medicine. 2005;80(2):176-82. 15671325 10.1097/00001888-200502000-00016

[28] Zwolsman S, te Pas E, Hooft L, Wieringa-de Waard M, van Dijk N Barriers to GPs' use of evidence-based medicine: a systematic review. Br J Gen Pract. 2012;62(600):e511-21. 22781999 10.3399/bjgp12X652382 PMC3381277

[29] Oude Rengerink K, Zwolsman SE, Ubbink DT, Mol BW, van Dijk N, Vermeulen H Tools to assess evidence-based practice behaviour among healthcare professionals. Evid Based Med. 2013;18(4):129-38. 23349216 10.1136/eb-2012-100969

[30] Te Pas E, van Dijk N, Bartelink ME, Wieringa-De Waard M Factors influencing the EBM behaviour of GP trainers: a mixed method study. Med Teach. 2013;35(3):e990-97. 23102157 10.3109/0142159X.2012.733044

[31] Geyer EM, Irish DE Isolated to integrated: an evolving medical informatics curriculum. Med Ref Serv Q. 2008;27(4):451-61. 19042724 10.1080/02763860802368324

[32] Kaneshiro KN, Emmett TW, London SK, et al Use of an audience response system in an evidence-based mini-curriculum. Med Ref Serv Q. 2008;27(3):284-301. 19042709 10.1080/02763860802198861

[33] O'Dwyer L, Kerns SC Evolution of an information literacy curriculum for third-year medical students. Med Ref Serv Q. 2011;30(3):221-32. 21800980 10.1080/02763869.2011.590411

[34] Tuttle BD, Von Isenburg M, Schardt C, Powers A PubMed instruction for medical students: searching for a better way. Med Ref Serv Q. 2009;28(3):199-210. 20183016 10.1080/02763860903069839

[35] Peterson LN, Rusticus SA, Wilson DA, Eva KW, Lovato CY Readiness for residency: A survey to evaluate undergraduate medical education programs. Academic Medicine. 2015;90(11):S36-42. 26505099 10.1097/ACM.0000000000000903

[36] D’Eon MF, Trinder K Evidence for the validity of grouped self-assessments in measuring the outcomes of educational programs. Evaluation & the health professions. 2014;37(4):457-69. 23396128 10.1177/0163278713475868

[37] Oxman AD, Sackett DL, Guyatt GH Users' guides to the medical literature. I. How to get started. The Evidence-Based Medicine Working Group. JAMA. 1993;270(17):2093-5. 8411577

[38] Aronoff SC, Evans B, Fleece D, Lyons P, Kaplan L, Rojas R Integrating evidence based medicine into undergraduate medical education: combining online instruction with clinical clerkships. Teach Learn Med. 2010;22(3):219-23. 20563945 10.1080/10401334.2010.488460

[39] Howard GS Response-shift bias: A problem in evaluating interventions with pre/post self-reports. Evaluation Review. 1980;4(1):93-106.

[40] Sastre EA, Denny JC, McCoy JA, McCoy AB, Spickard A 3rd Teaching evidence-based medicine: Impact on students' literature use and inpatient clinical documentation. Med Teach. 2011;33(6):e306-12. 21609166 10.3109/0142159X.2011.565827

[41] Nimon K, Zigarmi D, Allen J Measures of program effectiveness based on retrospective pretest data: are all created equal? American Journal of Evaluation. 2011;32(1):8-28.

